# Streptococcal protein SIC activates monocytes and induces inflammation

**DOI:** 10.1016/j.isci.2021.102339

**Published:** 2021-03-20

**Authors:** Ariane Neumann, Lotta Happonen, Christofer Karlsson, Wael Bahnan, Inga-Maria Frick, Lars Björck

**Affiliations:** 1 Division of Infection Medicine, Department of Clinical Sciences, BMC, Lund University, 22184, Lund, Sweden

**Keywords:** Immunology, Microbiology, Clinical Microbiology

## Abstract

*Streptococcus pyogenes* is a major bacterial pathogen in the human population and isolates of the clinically important M1 serotype secrete protein Streptococcal inhibitor of complement (SIC) known to interfere with human innate immunity. Here we find that SIC from M1 bacteria interacts with TLR2 and CD14 on monocytes leading to the activation of the NF-κB and p38 MAPK pathways and the release of several pro-inflammatory cytokines (e.g. TNFα and INFγ). In human plasma, SIC binds clusterin and histidine-rich glycoprotein, and whole plasma, and these two purified plasma proteins enhanced the activation of monocytes by SIC. Isolates of the M55 serotype secrete an SIC homolog, but this protein did not activate monocytes. M1 isolates are common in cases of invasive *S. pyogenes* infections characterized by massive inflammation, and the results of this study indicate that the pro-inflammatory property of SIC contributes to the pathology of these severe clinical conditions.

## Highlights

• M1 SIC, a secreted *S. pyogenes* protein interacts with CD14 and TLR2 on monocytes
• SIC triggers activation of NF-κB and p38 MAPK pathways and release of TNFα and INFγ
• Presence of human plasma enhanced SIC-mediated monocyte activation
• M55-SIC homolog has no effect on monocytes

## Graphical abstract

**Figure:**
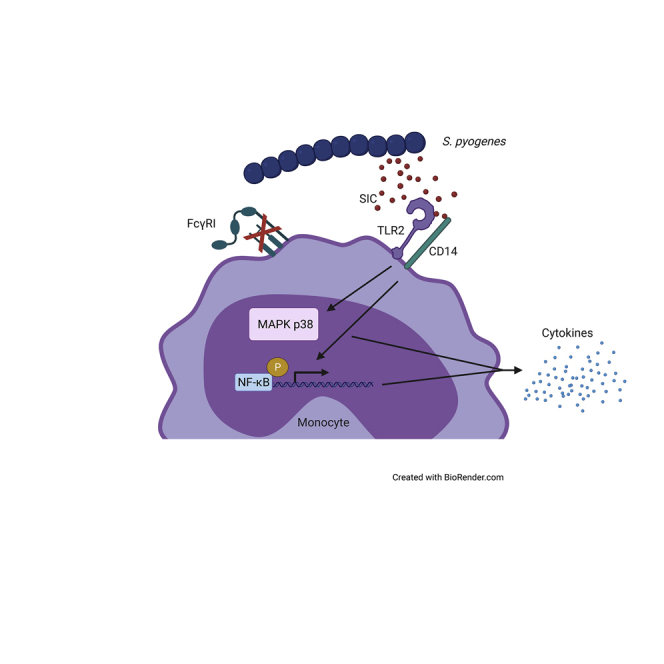

## Introduction

*Streptococcus pyogenes* is a significant pathogen causing a wide range of infections and post-infection sequelae in the human population (Carapetis et al., 2005). M protein is a virulence determinant and a fibrous surface protein in *S. pyogenes* [for more references, see (Cunningham, 2000)]. Based on sequence variation in the NH_2_-terminal surface-exposed tip of M protein, isolates of *S. pyogenes* are divided into more than 200 M serotypes, and since the 1980s strains of the M1 serotype have dominated worldwide and are frequently isolated from patients with severe invasive infections causing at least 150,000 deaths annually (Carapetis et al., 2005). The reason for the evolutionary success of the M1 serotype is not clear, but the production of Streptococcal inhibitor of complement (SIC) by M1 strains probably represents a contributing factor.

SIC is a secreted protein which was discovered and isolated from M1 bacteria and found to bind the plasma proteins clusterin and histidine-rich glycoprotein (HRG) and to interfere with the membrane attack complex of complement (Åkesson et al., 1996). The gene encoding SIC is highly polymorphic among M1 isolates and shows variation even during ongoing experimental infection in mice (Hoe et al., 1999), demonstrating a unique degree of adaptation to selective pressure. SIC enhances the ability of M1 bacteria to colonize the mouse mucosal surface after intranasal infection (Lukomski et al., 2000) and blocks the activity of antibacterial proteins and peptides (Fernie-King et al., 2002; Frick et al., 2003; Egesten et al., 2007) In addition, SIC promotes bacterial growth in human blood and virulence in a murine model of systemic infection (Pence et al., 2010). The protein modulates fibrinolysis resulting in enhanced bacterial survival in fibrin clots (Frick et al., 2018). SIC also inhibits the antibacterial activity of histones, and complexes between histones and SIC boost histone-triggered release of cytokines and chemokines in human blood (Westman et al., 2018), further underlining the multiple effects of SIC on innate immunity and the host-bacteria relationship.

Circulating monocytes play an important role in host immune defense against bacterial pathogens. They are rapidly recruited to sites of inflammation and infection where they can differentiate into macrophages and dendritic cells (DCs) or become effector cells with distinct antimicrobial activity (Serbina et al., 2008). Undifferentiated monocytes secrete cytokines when activated by microbial components and following phagocytosis of microbes (Ghebrehiwet et al., 2014); and for this purpose, monocytes carry a broad range of receptors on their surface, which downstream activate various signaling pathways. These microbial pattern-recognition receptors (PRRs) play an important role detecting pathogen-associated molecular patterns, leading to the activation of monocytes and other immune cells (Dale et al., 2008). Toll-like receptors (TLRs) are type 1 membrane proteins with an extracellular leucine-rich domain (Kawasaki and Kawai, 2014), constituting an essential class of PRRs. Within the TLR superfamily, TLR2 has been associated with the recognition of Gram-positive bacteria and their derived products, like M1 protein (Ozinsky et al., 2000; Henneke et al., 2001; Joosten et al., 2003; Påhlman et al., 2006; Wu et al., 2016). The activity of TLR2 in response to bacterial products can be elevated by the interaction between TLR2 and glycophosphatidylinositol (GPI)-anchored CD14 (Schwandner et al., 1999; Yoshimura et al., 1999), which for instance acts as a co-receptor for LPS and peptidoglycan (Henneke et al., 2001). Signaling via TLR2 might lead to downstream activation of NF-κB (Kawai and Akira, 2007) and further cytokine secretion.

Patients with *S. pyogenes* sepsis and septic shock display high cytokine levels in plasma (Cavaillon et al., 2003), which raised the central question of the present study; could SIC, secreted by M1 streptococci, activate monocytes and contribute to the life-threatening inflammation in these conditions. The results show that SIC by binding to TLR2 and CD14 at the monocyte surface activates the NF-κB and p38 MAPK pathways resulting in the release of pro-inflammatory cytokines. The observation that this activity of SIC was enhanced in the presence of plasma or purified plasma proteins known to form complexes with SIC, further support the notion that SIC is an important virulence factor in severe *S. pyogenes* infection.

## Results

### SIC in bacterial growth medium triggers activation of NF-κB and p38 MAP kinase

**Figure 1:**
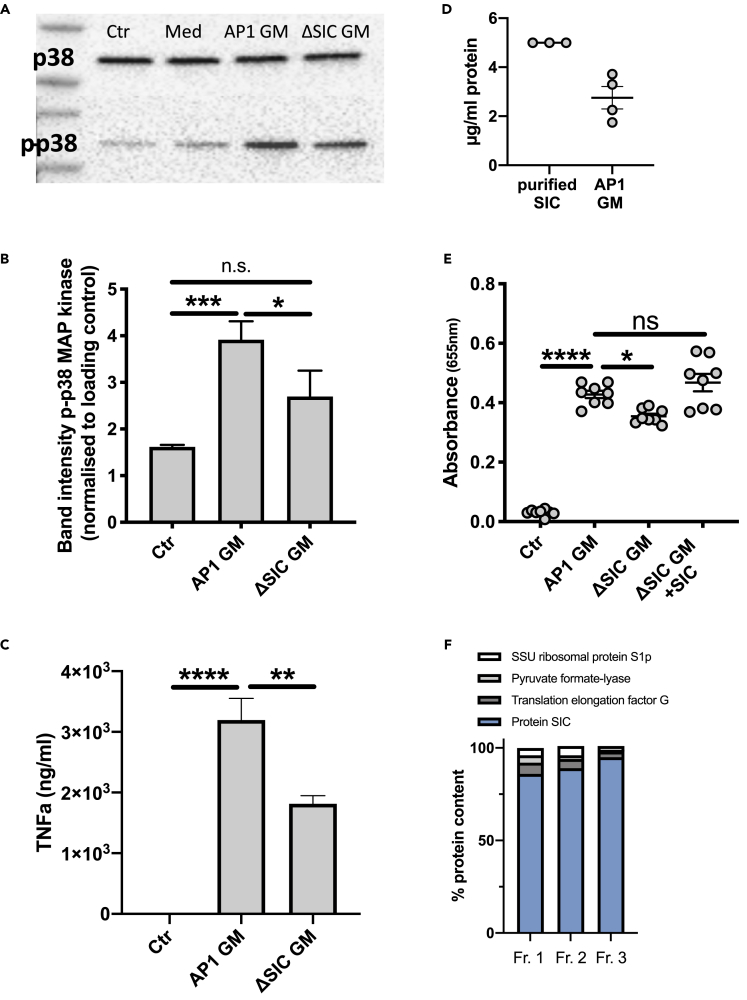
Growth medium from S. pyogenes secreting SIC activates THP1 cells (A) Lysates of THP1 cells after incubation with AP1 or ΔSIC growth medium (GM) were subjected to SDS-PAGE and Western blot. The membranes were probed with antibodies against p38 MAP kinase (p38) and phospho-p38 MAP kinase (p-p38). Ctr: medium. Med: concentrated medium. (B) Band intensity analysis of the Western blot depicted in A. The band intensity of phosphorylated p38 was normalized to the band intensity of p38 MAPK. (C) TNFα release from THP1 cells incubated with GM from AP1 or ΔSIC was measured by ELISA. (D) The SIC concentration in AP1 GM was determined by direct ELISA and compared to purified protein. (E) THP1 cells were incubated for 18 hr with GM from AP1, ΔSIC or ΔSIC +3 μg/mL purified SIC. Absorbance of QuantiBlue color change as indicator for NF-κB activation was measured at 655 nm. (F) Quantitative MS analysis of purified SIC. Three fractions of purified SIC were digested with trypsin and analyzed with mass spectrometry. The resulting data was searched against the *S. pyogenes* AP1 proteome and the identified peptides were further analyzed with MS1 precursor intensity-based quantification. The figure shows the top 4 most abundant streptococcal proteins identified in the fractions. The y axis is relative percentage of top4 protein intensity. All data represent mean ± SEM of 3 independent experiments, one-way ANOVA, Dunnett's multiple comparison test, with single pooled variance. ∗p < 0.05, ∗∗p < 0.01, ∗∗∗p < 0.001, ∗∗∗∗p < 0.0001.

Supernatants from streptococcal cultures were tested on the monocyte THP1 cell line. *S. pyogenes* strains AP1 and ΔSIC were grown in RPMI +10% FBS and growth media (GM) were collected and added to THP1 cells. The GM from both bacterial strains clearly mediated phosphorylation of the p38 MAP kinase as compared to medium alone (Figures 1A and 1B). However, when band intensities were normalized to the loading control, the GM from the ΔSIC strain was significantly less efficient in activating the MAP kinase (Figure 1B). The release of TNFα was significantly lower when THP1 cells were incubated with the ΔSIC GM as compared to AP1 GM (Figure 1C). Next, the impact of *S. pyogenes* GM on nuclear factor 'kappa-light-chain-enhancer' of activated B-cells, NF-κB activation, was analyzed; GM from AP1 triggered the activation, which was only slightly but still significantly higher compared to when THP1 cells were incubated with GM from ΔSIC bacteria (Figure 1E). A direct ELISA was performed to determine the concentration of SIC in the GM from AP1 bacteria. Purified SIC protein at a concentration of 5 μg/mL served as a control (Figure 1D), and the GM from the *S. pyogenes* AP1 strain was found to contain around 3 μg SIC/ml (Figure 1D). When SIC was added to the GM from ΔSIC bacteria to obtain a concentration of 3 μg/mL, NF-κB activation increased to levels similarly to that of AP1 GM (Figure 1E), indicating that SIC produced and secreted during bacterial growth is responsible for the activation of THP1 cells. Purified SIC was analyzed by quantitative mass spectrometry (MS), revealing an SIC protein content of over 90% in 3 different fractions (Figure 1F).

### SIC influences the release of various pro-inflammatory cytokines

**Figure 2:**
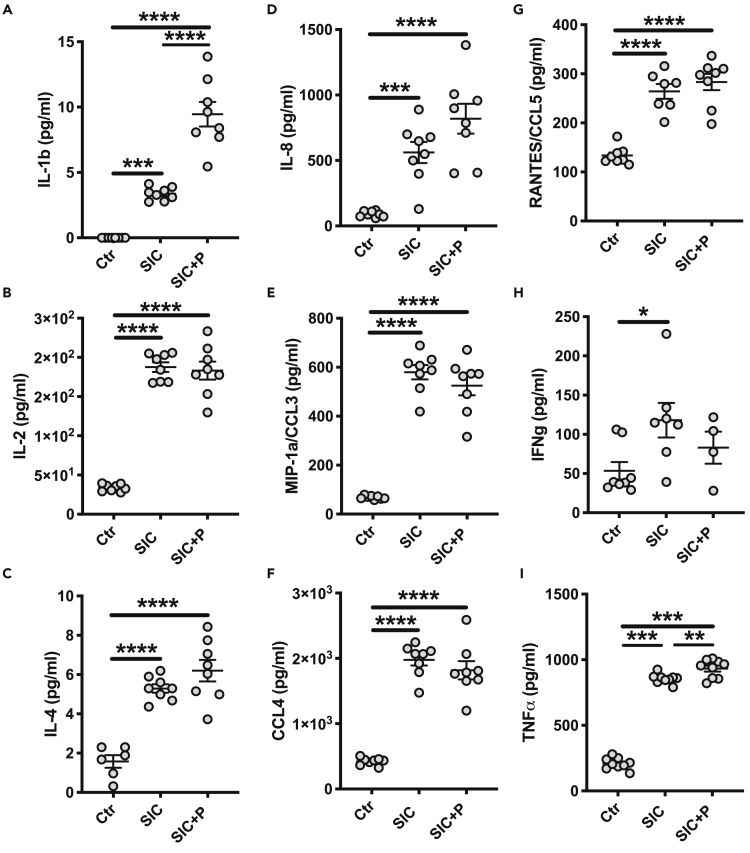
SIC changes the cytokine profile of THP1 cells (A–I) Cells were incubated for 2 hr with 5 μg/mL SIC +/− 2.5% plasma. Supernatants were collected and analyzed using a 27-plex cytokine assay. All data represent mean ± SEM of 3 independent experiments, one-way ANOVA, Dunnett's multiple comparison test, with single pooled variance. ∗p < 0.05, ∗∗∗p < 0.001, ∗∗∗∗p < 0.0001.

When AP1 bacteria is grown to early stationary phase the concentration of SIC in the GM reaches 5 μg/mL (Frick et al., 2003), and this concentration was used in the following experiments. Apart from monocytes, we wanted to analyze the effect of SIC also on the nasopharyngeal epithelial cell line Detroit 562. To exclude cytotoxic effects of SIC on the cells, LDH release was measured (Figure S1A). No detrimental effect was observed in either of the cell types when incubated for 18 hr with 5 μg/mL of SIC in the presence or absence of plasma (Figure S1A). Subsequently, THP1 monocytes and Detroit cells were incubated with SIC alone or in the presence of plasma for 2 hr, and the supernatants were collected and analyzed for cytokine content (Figures 2A–2I). Detroit 562 cells showed no upregulation or downregulation in response to SIC alone or in combination with plasma (data not shown). In contrast, incubation of THP1 cells with SIC caused a significant release of several pro-inflammatory cytokines like IL-1b, IL-2, IL-8, and TNFα (Figures 2A, 2B, 2D, and 2I). SIC-induced cytokine release was significantly enhanced by plasma in case of IL-1b (Figure 2A) and TNFα (Figure 2I), while plasma presence slightly reduced the SIC-mediated cytokine release for IL-2, MIP-1a/CCL3, IFNg, and CCL4 (Figures 2B, 2E, 2F, and 2H).

### SIC mediates p38 MAP kinase phosphorylation and triggers NF-κB activation in THP1 monocytes

**Figure 3:**
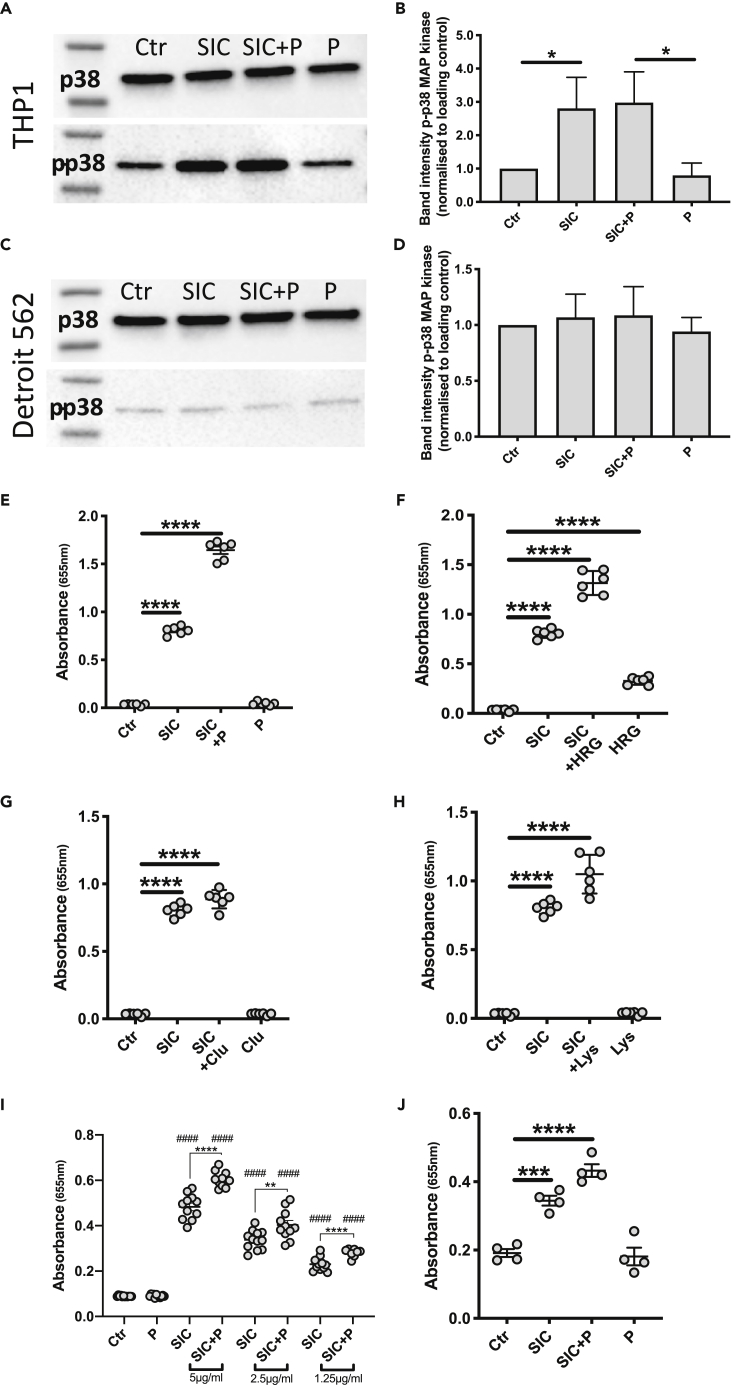
SIC phosphorylates p38 MAP kinase in THP1 cells and activates NF-κB (A and C) Representative images of Western blot analysis of cell extracts from THP1 and Detroit 562 cells incubated with 5 μg/mL SIC +/− 2.5% plasma (P). Membranes were incubated with anti-p38 MAPK (p38) and anti-phospho-p38 MAPK (p-p38) antibodies. (B and D) Band intensity analysis of Western blots displayed in A and C. The band intensity of phosphorylated p38 was normalized to the band intensity of p38 MAPK. (E–H) Cells were incubated with 5 μg/mL SIC alone or in combination with E: 2.5% plasma F: 200 ng/mL HRG G: 5 μg/mL clusterin H: 5 μg/mL lysozyme for 18 hr. Cells were also incubated with plasma and the plasma proteins alone. (I) THP1 cells were incubated with 5 μg/mL, 2.5 μg/mL or 1.25 μg/mL SIC in the presence or absence of 2.5% plasma (P) for 18 hr. (J) THP1 cells were differentiated into macrophages with 100 nM PMA and the activation of NF-κB was analyzed after incubation with 5 μg/mL SIC +/− 2.5% plasma (P). NF-κB activation was detected as color changes of QuantiBlue solution, absorbance was measured at 655 nm. All data represent mean ± SEM of 3-5 independent experiments, one-way ANOVA, Dunnett's multiple comparison test, with single pooled variance. ∗p < 0.05, ∗∗∗p < 0.001, ∗∗∗∗p < 0.0001. ####p < 0.0001 compared to untreated control.

Next, possible downstream effects of TNFα release and other cytokines was investigated. Secretion of TNFα and its subsequent binding to a cell surface receptor is involved in p38 MAP kinase activation and phosphorylation (Liu et al., 2000). This activation in turn leads to a secondary release of cytokines including TNFα. Thus, THP1 and Detroit cells were incubated with SIC in the presence and absence of plasma and cell lysates were analyzed by Western blotting (Figures 3A–3D). Phosphorylation of p38 MAP kinase in THP1 cells was mediated by SIC alone or by SIC in plasma (Figures 3A and 3B), whereas no increased phosphorylation was observed in the Detroit cells (Figures 3C and 3D). The phosphorylation of p38 MAP kinase can influence activation of another key player in regulating immune responses to infections, NF-κB (Saha et al., 2007). Additionally, the secretion of TNFα and the binding to its receptor triggers the activation of NF-κB (Hsu et al., 1995), and to further study the impact of SIC on THP1 cells, THP1-Blue NF-κB cells were zutilized. With these cells, the activation of NF-κB can be monitored by zanalyzing the expression of secreted embryonic alkaline phosphatase (SEAP). The cells were incubated with SIC alone or with SIC together with whole plasma or purified plasma proteins (clusterin, lysozyme and HRG) for 18 hr and the supernatants were analyzed. Previous studies have shown that SIC binds clusterin and HRG in plasma (Åkesson et al., 1996) and inactivates the antibacterial activity of lysozyme (Fernie-King et al., 2002). Again, no cytotoxic effect, measured by release of LDH, was observed with any of the stimuli (data not shown). Next, the expression of SEAP was measured and in all experimental set ups SIC alone significantly triggered NF-κB activation (Figures 3E–3H). The addition of plasma or the three purified plasma proteins known to interact with SIC enhanced the activation triggered by SIC, whereas plasma, clusterin, and lysozyme alone had no effect on the activation of NF-κB (Figures 3E, 3G, and 3H). Only HRG exerted a low but significant activation of NF-κB (Figure 3F). To observe what concentrations of SIC are able to trigger NF-κB activation in THP1 monocytes, a dose-dependent response was analyzed, incubating the cells with 5 μg/mL, 2.5 μg/mL and 1.25 μg/mL SIC. While the NF-κB activation visibly decreased with lower concentrations, the lowest tested concentration of SIC (1.25 μg/mL) was still able to cause a significant response (Figure 3I).

Monocytes differentiate into macrophages or DCs after leaving the blood stream which may lead to changes in their receptor profile (Gantner et al., 1997), and the effect of SIC on NF-κB activation was therefore analyzed in PMA-differentiated THP1 cells (Figure 3J). As seen before with the monocytic THP1 cells, SIC alone and in combination with plasma significantly triggered a strong NF-κB activation also after PMA-induced differentiation.

### The central region of SIC is responsible for the activation of NF-κB and p38 MAP kinase

**Figure 4:**
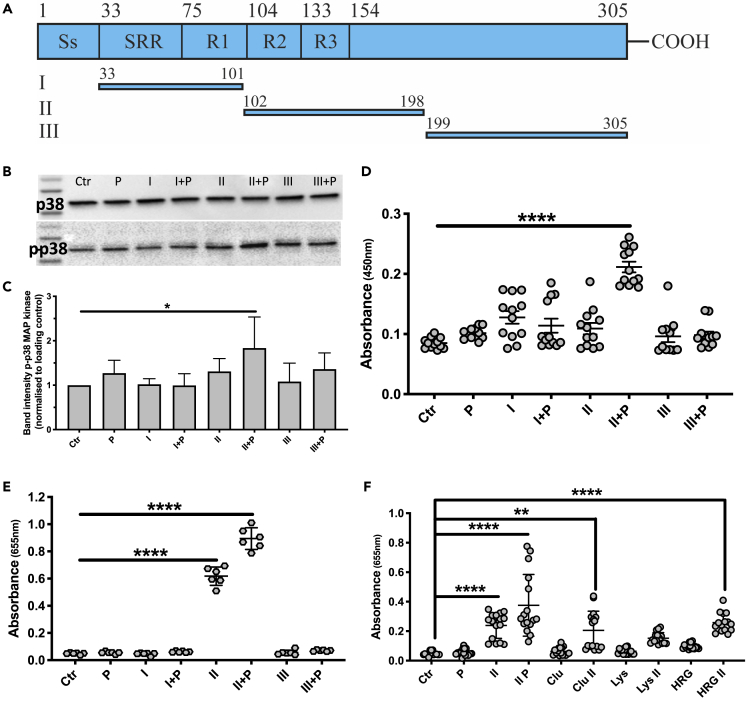
Fragment II of SIC (aa 102–198) is responsible for the activation of THP1 cells (A) Schematic structure of SIC from strain AP1 and zlocalization of the three fragments used. The signal sequence (Ss) is cleaved off during maturation. The mature secreted protein contains a short repeat region (SRR), as well as three tandem repeats (R1-R3). The numbers indicate amino acid positions, and also refer to the length of each of the SIC fragments (I-III), which are recombinantly expressed in *E. coli*. Based on previous publications (Fernie-King et al., 2004; Binks et al., 2005), the sizes of the fragments were chosen. (B) Western blot analyses of THP1 cell lysates after stimulation with 5 μg/mL SIC fragments (I, II, III) +/− 2.5% plasma (P). Membranes were incubated with antibodies against p38 MAPK (p38) and phosphorylated p38 MAPK (p-p38). (C) Quantification of phosphorylation of Western blots depicted in B. The band intensities of p-p38 samples were normalized to loading control (p38 band) and values analyzed. (D) THP1 cells were incubated with 5 μg/mL SIC fragments +/− 2.5% plasma (P) and the release of TNFα was measured by ELISA. (E) Activation of NF-κB by SIC fragments +/− plasma was analyzed by detecting QuantiBlue color change at 655 nm. (F) Absorbance of QuantiBlue color change at 655 nm, as indicator of NF-κB activation after THP1 cell incubation with 5 μg/mL SIC fragment II alone and in combination with 2.5% whole plasma (P), 5 μg/mL clusterin (Clu), 5 μg/mL lysozyme (Lys) or 200 ng/mL HRG. All data represent mean ± SEM of 4-6 independent experiments, one-way ANOVA, Dunnett's multiple comparison test, with single pooled variance. ∗p < 0.05, ∗∗p < 0.01, ∗∗∗∗p < 0.0001.

To map the region of SIC responsible for the observed activation of THP1 cells, three recombinant fragments of SIC were expressed and utilized (Fernie-King et al., 2004; Frick et al., 2018) The fragments span the mature secreted SIC protein (amino acids 1–33 correspond to the signal sequence): fragment I covers amino acids 33–101, fragment II aa 102–198, and fragment III aa 199–305 (Figure 4A). The combination of fragment II and plasma was found to mediate phosphorylation of p38 MAP kinase (Figures 4B and 4C). As mentioned, phosphorylation of p38 MAP kinase may result in secretion of pro-inflammatory cytokines like TNFα, and similar to the effect observed for the p38 MAP kinase, fragment II in combination with plasma triggered a significant release of TNFα (Figure 4D). A strong activation of NF-κB was also detected in response to fragment II alone and in the presence of plasma (Figure 4E). To investigate whether the plasma proteins known to interact with SIC were responsible for the enhanced effect of fragment II in combination with plasma, THP1 cells were incubated with fragment II together with the purified plasma proteins, as described above, and SEAP expression was analyzed. In combination with fragment II, clusterin and HRG, but not lysozyme, significantly triggered NF-κB activation (Figure 4F).

### SIC interacts with TLR2 and CD14 on THP1 cells

**Figure 5:**
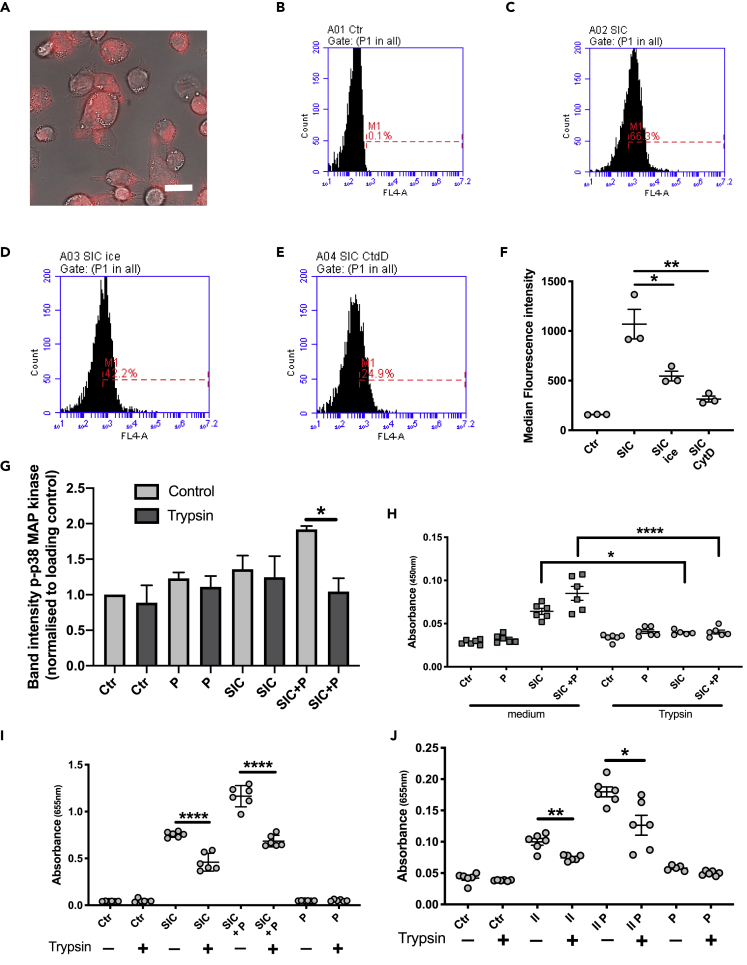
Analysis of the interaction between SIC and THP1 cells (A) THP1 cells were incubated for 45 min with 5 μg/mL AlexaFluor 633-labeled SIC protein (red). Live imaging of the cells was used to observe the interaction of SIC with the THP1 cells. Scale bar represents 20 μm. (B–E) Flow cytometry analysis of SIC-monocyte interaction. B: THP1 cells incubated with medium (Ctr) C: THP1 cells incubated with 5 μg/mL SIC (SIC) D: THP1 cells incubated with 5 μg/mL SIC on ice (SIC ice) E: THP1 cells pre-incubated with 20 μM CytD for 30 min, then incubated with 5 μg/mL SIC (SIC CtdD). (F) Median fluorescent intensity analysis of SIC interaction with the cells in B-E. Data shown from 3 independent experiments, ∗p < 0.05, ∗∗p < 0.01. (G) THP1 cells, untreated or trypsinized, were incubated with 5 μg/mL SIC +/− 2.5% plasma (P). Lysates of the cells were subjected to SDS-PAGE analysis and Western blot. Membranes were probed with antibodies against p38 MAPK (p38) and phosphorylated p38 MAPK (p-p38). Band intensities were analyzed. (H) TNFα secretion by untreated and trypsinized THP1 cells, incubated with 5 μg/mL by SIC +/− 2.5% plasma (P) was measured by ELISA at 450 nm. (I) Untreated and trypsinized THP1 cells were incubated with 5 μg/mL SIC +/− 2.5% plasma (P) and activation of NF-κB was measured at 655 nm. (J) Untreated and trypsinized THP1 cells were incubated with 5 μg/mL SIC fragment II +/− 2.5% plasma (P) and activation of NF-κB was measured. All data represent mean ± SEM of 3-5 independent experiments, one-way ANOVA, Dunnett's multiple comparison test, with single pooled variance ∗p < 0.05, ∗∗p < 0.01, ∗∗∗∗p < 0.0001.

The demonstrated interaction between SIC and monocytes raised the question whether SIC, similarly to M1 protein (Valderrama et al., 2017), could be taken up by monocytes. Indeed, live imaging of THP1 cells incubated with AlexaFluor 633-labeled SIC protein, showed a pattern indicating surface binding and/or cellular uptake of the protein (Figure 5A). To quantify the uptake/binding, flow cytometry analyses were performed (Figures 5B–5F). As compared to the control, the number of cells positive for SIC suggested binding or uptake of the protein (Figures 5B, 5C, and 5F). Incubation of the cells on ice significantly reduced uptake/binding of SIC (Figures 5D and 5F), whereas pre-incubation with cytochalasin D almost completely abolished uptake/binding (Figures 5E and 5F). The results indicated an interaction between SIC and monocyte surface receptors.

**Figure 6:**
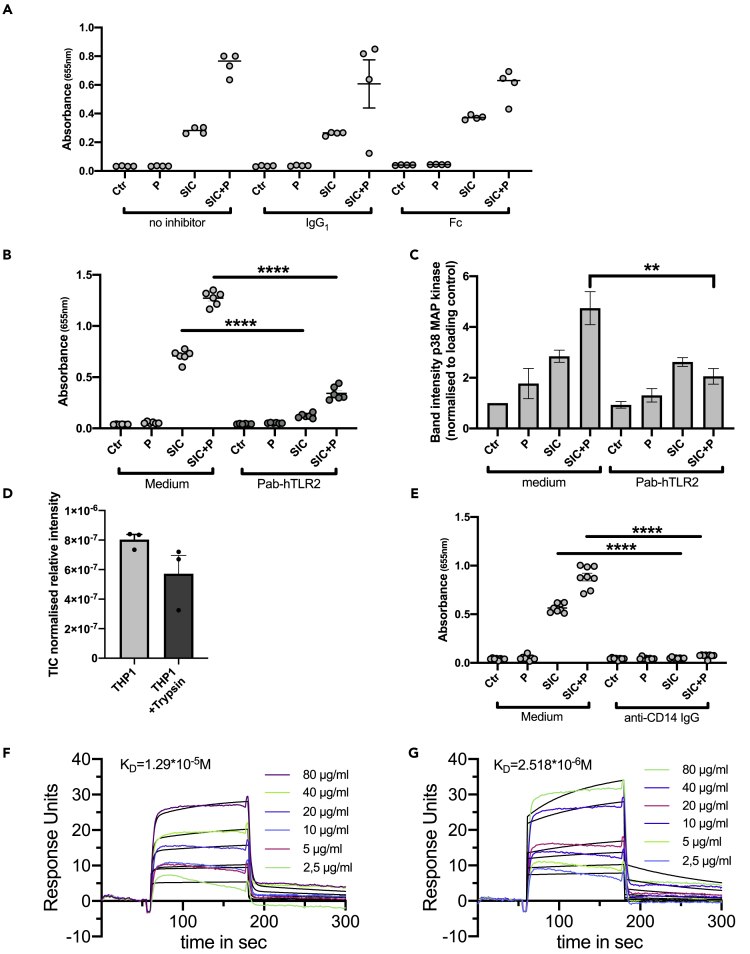
SIC interacts with TLR2 and CD14 to activate THP1 cells (A) THP1 cells were pre-incubated for 30 min with IgG_1_ and Fc fragment and then incubated with 5 μg/mL SIC +/− 2.5% plasma (P). NF-κB activation was analyzed at 655 nm. (B) Cells were pre-incubated with a neutralizing antibody against TLR2 and then incubated with 5 μg/mL SIC +/− 2.5% plasma (P). Activation of NF-κB was measured at 655 nm. (C) Quantification of phosphorylation of Western blots of THP1 cells treated with a neutralizing antibody against TLR2. The band intensities of p-p38 samples were normalized to loading control (p38 band) and values analyzed. (D) Quantitative DIA-MS analysis of THP1 cells before and after trypsin treatment. (E) THP1 cells were pre-incubated with antibodies against CD14 and then incubated with 5 μg/mL SIC +/− 2.5% plasma (P). NF-κB activation was analyzed. (F) Surface plasmon resonance data show interaction of different SIC concentrations to immobilized TLR2. Representative original data are displayed in colors, 1:1 Langmuir fitted curves are presented in black. (G) Surface plasmon resonance data show interaction of different SIC concentrations to immobilized CD14. Representative original data are displayed in colors, 1:1 Langmuir fitted curves are presented in black. All data represent mean ± SEM of 3 independent experiments, one-way ANOVA, Dunnett's multiple comparison test, with single pooled variance. ∗∗p < 0.01, ∗∗∗∗p < 0.0001.

Monocytes, like macrophages and neutrophils, express various receptors on their surface to zrecognize invading pathogens, e.g. TLR2 or FcγRI (CD64) (Juarez et al., 2010; Bournazos et al., 2016). To remove all surface proteins the THP1 cells were pre-treated with trypsin, and after incubation with SIC in the presence and absence of plasma, cell lysates were analyzed by Western Blotting for p38 MAPK phosphorylation. Trypsin treatment reduced the phosphorylation of p38 MAPK mediated by SIC in the presence of plasma (Figure 5G) and significantly reduced the release of TNFα (Figure 5H), as well as the activation of NF-κB by SIC in the presence and absence of plasma (Figure 5I). The reduction in monocyte responses following trypsin digestion of surface proteins was also recorded for fragment II of SIC in the presence and absence of plasma (Figure 5J). To investigate whether FcγRI (CD64) might be an interaction partner of SIC, the effect of blocking the Fc receptor with IgG and IgG Fc fragments was analyzed. Pre-treatment of the cells with the FcγRI ligands had no impact on SIC-mediated NF-κB activation (Figure 6A). *S. pyogenes* bacteria as well as M1 protein are known to bind to TLR2 on host cells surfaces (Sigurdardottir et al., 2010). Dimerization of TLR2 with a receptor interaction partner induces a signaling cascade involving NF-κB activation and subsequent cytokine release (Shmuel-Galia et al., 2016). To explore the possibility that the binding of SIC to TLR2 leads to activation of NF-κB, this pathway was blocked with a specific antibody against TLR2 (Figure 6B). Pre-treatment with this antibody was found to significantly decrease the activation of THP1 cells when incubated with SIC, both in the presence or absence of plasma (Figure 6B). This was further shown by a reduced phosphorylation of p38 MAP kinase when TLR2 was blocked, in the case of SIC + P (Figure 6C). Mass spectrometry analysis of THP1 cells treated with or without trypsin revealed a decrease of TLR2 peptides in the trypsin treated samples (Figure 6D). It has been reported that macrophages also can be activated by bacterial peptidoglycan in a CD14-dependent fashion (Gupta et al., 1996), and that the interaction of CD14 with TLR2 can lead to a synergistically enhanced activation, triggered by Gram-positive bacteria (Yoshimura et al., 1999). Additionally, Henneke et al. suggested that TLR2 and CD14 act as co-receptors for Group B streptococcal secreted products (Henneke et al., 2001). With this background, the impact of CD14 on SIC-mediated monocyte activation was investigated and blocking of the receptor with specific antibodies completely abolished SIC-mediated NF-κB activation (Figure 6E).

To investigate whether SIC directly interacts with these host surface receptors, we performed surface plasmon resonance analysis. In this set of experiments, we immobilized either TLR2 or CD14 (ligands) on a CM5 chip and used different SIC concentrations as analyte. Binding studies of SIC generated K_D_ values of 1.29 x 10^−5^M for TLR2 (Figure 6F) and 2.518x10^−6^M for CD14 (Figure 6G), showing affinity of SIC for both TLR2 and CD14.

### SIC activates primary CD14 + monocytes in a TLR2-dependent fashion

**Figure 7:**
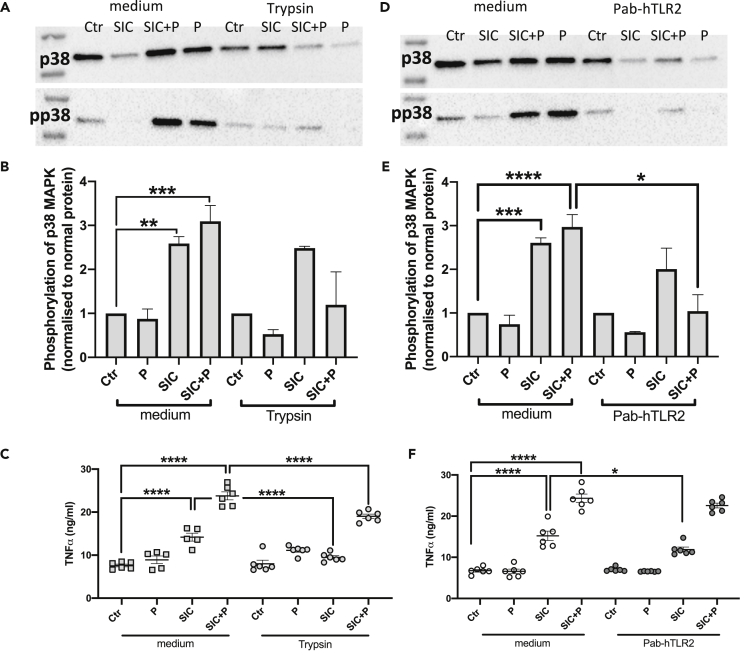
SIC triggers p38 MAPK activation and TNFα release from primary CD14 + monocytes (A) Western blot analyses of CD14^+^ cell lysates before and after trypsin treatment (100 μg/mL); cells were stimulated with 5 μg/mL SIC +/− 2.5% plasma (P). Membranes were incubated with antibodies against p38 MAPK (p38) and phosphorylated p38 MAPK (p-p38). (B) Quantification of phosphorylation of Western blots of CD14^+^ monocytes treated with 100 μg/mL trypsin for 30 min. The band intensities of p-p38 samples were normalized to loading control (p38 band) and values analyzed. (C) CD14^+^ cells were pre-incubated with 100 μg/mL trypsin, and then incubated with 5 μg/mL by SIC +/− 2.5% plasma (P). Absorbance was measured by ELISA at 450 nm. (D) Western blot analyses of CD14^+^ cell lysates after stimulation with 5 μg/mL SIC +/− 2.5% plasma (P). Cells were pre-incubated with Pab-hTLR2 for 20 min. Membranes were incubated with antibodies against p38 MAPK (p38) and phosphorylated p38 MAPK (p-p38). (E) Quantification of phosphorylation of Western blots of CD14^+^ cells treated with a neutralizing antibody against TLR2. The band intensities of p-p38 samples were normalized to loading control (p38 band) and values analyzed. (F) CD14^+^ cells were pre-incubated with a neutralizing antibody against TLR2, and then incubated with 5 μg/mL SIC +/− 2.5% plasma (P). Release of TNF α was measured at 450 nm. All data represent mean ± SEM of 3-5 independent experiments, one-way ANOVA, Dunnett's multiple comparison test, with single pooled variance. ∗p < 0.05, ∗∗p < 0.01, ∗∗∗p < 0.001 ∗∗∗∗p < 0.0001.

Finding that SIC from *S. pyogenes* activated THP1 monocytes, acute monocytic leukemia cells, we sought to investigate the effect of SIC on primary blood derived monocytes. We therefore purified CD14^+^ monocytes from leukocyte concentrate via density gradient centrifugation and magnetic bead labeling. Similar to the results observed for the THP1 cells, a pre-incubation for 30 min with trypsin strongly reduced the phosphorylation of p38 MAPK triggered by SIC in the presence of plasma (Figures 7A and 7B). Incubation of CD14^+^ cells with SIC significantly triggered the secretion of TNFa (Figure 7C), which was significantly diminished by the pre-treatment of the cells with trypsin (Figure 7C). To test whether trypsin treatment has any detrimental effect on the cells, LDH release was measured. The 30 min pre-incubation of CD14^+^ cells with 100 μg/mL trypsin had no effect on the secretion of LDH (Supp. Figure 1B). To analyze a possible involvement of TLR2, the primary cells were incubated with the neutralizing antibody Pab-hTLR. Here, the block of TLR2 significantly reduced the phosphorylation of p38 MAPK mediated by SIC in the presence of plasma (Figures 7D and 7E). Additionally, the TNFa release triggered by SIC was significantly diminished by the neutralizing antibody (Figure 7F).

### Only M1-SIC triggers immune responses in THP1 monocytes

**Figure 8:**
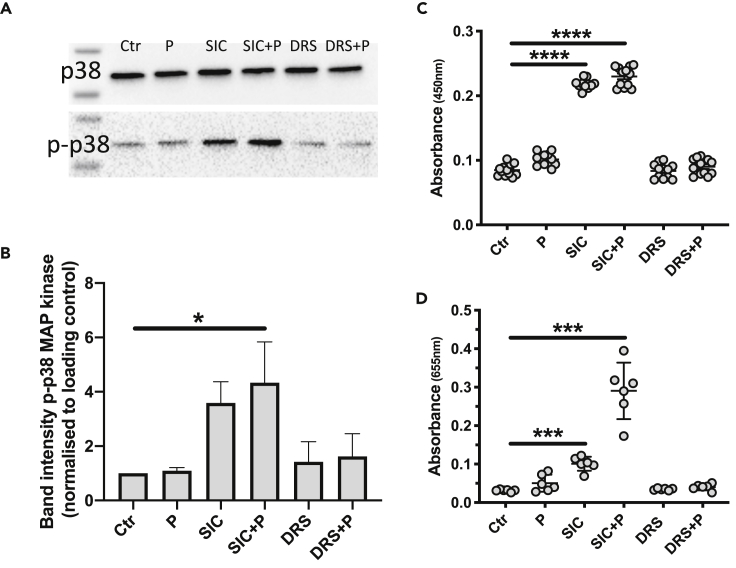
An M55-SIC homolog shows no affinity for clusterin and HRG and does not activate THP1 cells (A) Lysates of THP1 cells incubated with 5 μg SIC/ml or DRS +/− 2.5% plasma (P) were subjected to SDS-PAGE analysis and Western blotting. Membranes were probed with antibodies against p38 MAPK (p38) and phosphorylated p38 MAPK (p-p38). (B) Band intensities of the Western blot in B were analyzed. (C) Release of TNFα from THP1 cells incubated with SIC or DRS in presence and absence of plasma (P). Absorbance detected at 450 nm. (D) Activation of NF-κB in cells incubated with 5 μg SIC/ml or DRS in presence and absence of 2.5% plasma (P). Absorbance was detected at 655 nm. All data represent mean ± SEM of 3-4 independent experiments, one-way ANOVA, Dunnett's multiple comparison test, with single pooled variance. ∗p < 0.05, ∗∗∗p < 0.001, ∗∗∗∗p < 0.0001.

Distantly related SIC (DRS) homologs are produced by *S. pyogenes* strains of M serotypes 12 and 55 (Hartas and Sriprakash, 1999) (Hartas and Sriprakash, 1999)[36] and similar to SIC from M1 isolates these homologs bind complement components and also compete with SIC for this binding, but in contrast to SIC they do not activate complement (Binks and Sriprakash, 2004). To investigate if a DRS had similar effects on monocytes as M1-SIC, DRS was purified from the growth medium of an *S. pyogenes* M55 strain. The purified DRS showed no cytotoxicity on THP1 cells as judged by LDH release (Figure S1C).When the effect of DRS on phosphorylation of p38 MAP kinase, release of TNFα and NF-κB activation was compared to SIC, DRS had no impact, neither alone nor in the presence of plasma, on the phosphorylation of p38 MAPK (Figures 8A and 8B) or the release of TNFα and NF-κB activation (Figures 8C and 8D).

## Discussion

In this investigation (the main findings are summarized in the graphical abstract) SIC was found to bind to TLR2/CD14 at the surface of THP1 monocytes and primary CD14^+^ monocytes resulting in the activation of the cells and the release of pro-inflammatory cytokines. SIC is known to bind clusterin and HRG in human plasma, and the presence of whole plasma or these individual plasma proteins, enhanced the activation, indicating that SIC-driven cytokine release could be particularly relevant in blood. This notion is further supported by the finding that a SIC homolog from a strain of the M55 serotype showed no pro-inflammatory activity. It is also noteworthy that the M12 and M55 serotypes are associated with post-streptococcal glomerulonephritis (Ferrieri et al., 1970; Dillon, 1979), an important sequelae of *S. pyogenes* infection, but not with invasive disease. The activation of NF-κB and MAPK in macrophages/monocytes by *S. pyogenes* (Wu et al., 2016) leads to the production of pro-inflammatory cytokines like TNFα in a MyD88-dependent fashion (Gratz et al., 2008). Similar to our finding that SIC-containing bacterial growth medium triggered activation of both the NF-κB and p38 MAPK pathways, growth medium of other Gram-positive bacteria, like *S. aureus*, triggered the release of antimicrobial peptides via the same pathways (Zhu et al., 2013).

*S. pyogenes* are internalized by macrophages where they replicate (O'Neill et al., 2016), and trigger inflammation (Valderrama et al., 2017). M1 protein has been discussed as a possible invasion factor, facilitating the uptake of the bacteria (Dombek et al., 1999; Ochel et al., 2014). Furthermore, the internalization of *S. pyogenes* has been reported to involve actin polymerization, with F-actin associated with intracellular streptococci (Dombek et al., 1999; Nobbs et al., 2007). Phagocytic activity, and thus the uptake of bacteria, is inhibited by cytochalasin D (Elliott and Winn, 1986; Kapellos et al., 2016). In relation to this work, it is interesting that Hoe and colleagues found that SIC was internalized by lung epithelial cells within few minutes, and the authors suggested that SIC interacts with intracellular proteins (Hoe et al., 2002). This study showed that SIC was taken up by the monocytes, an uptake that was inhibited by cytochalasin D and incubation on ice, indicating that SIC may interact with proteins involved in the cytoskeletal rearrangement, similar to whole streptococci of the M1 serotype (Dombek et al., 1999).

In addition to a possible intracellular interaction with cytoskeletal proteins, SIC was demonstrated to interact with monocyte surface receptors TLR2 and CD14. CD14, a GPI-anchored protein, is expressed by myeloid cells, like monocytes, macrophages, and neutrophils (Van Amersfoort and Kuiper, 2007) The relatively low expression levels of this receptor is increased by stimulation with LPS or DMSO (Zamani et al., 2013), and CD14 is hypothesized to facilitate the uptake of LPS into macrophages (Ding et al., 1999). Since CD14 lacks a transmembrane signaling domain (Van Amersfoort and Kuiper, 2007), it often interacts with another accessory surface receptor, e.g. a TLRs. TLR2 is associated with the recognition of Gram-positive bacteria and their lipoproteins; lipoproteins are the major ligand for this receptor (Oliveira-Nascimento et al., 2012). M1 protein activates human peripheral monocytes via TLR2 interaction, leading to IL-6 secretion (Påhlman et al., 2006). However, M1 might not be a direct TLR agonist, but rather operating through interactions with other secreted streptococcal products (Valderrama et al., 2017), triggering the subsequent cytokine release. Similar to CD14, the expression of TLR2 can be induced by bacterial products and whole bacteria, like *S. pyogenes* (Wu et al., 2016). However, the primary interaction of the bacterial product can be with CD14 followed by the involvement of a TLR (Van Amersfoort and Kuiper, 2007). The data of this study indicate that this could be valid for SIC, and that TLR2 and CD14 act as co-receptors for SIC. Based on these findings, we zhypothesize that the interaction of SIC with the surface receptors, rather than the uptake into the cells might lead to the signaling and activation of the NF-kB and p38 MAPK pathways.

Invasive *S. pyogenes* infections, particularly streptococcal toxic shock syndrome and necrotizing fasciitis, are feared clinical conditions, and the prevalence of invasive diseases has been reported to be at least 663,000 cases annually worldwide with fatality rates around 25 percent. However, the large majority of *S. pyogenes* diseases are relatively uncomplicated superficial infections of the throat (pharyngitis) and skin (impetigo and erysipelas) with 700 million estimated cases per year (Carapetis et al., 2005). In addition, children and youngsters are often healthy carriers of the bacterium in the pharynx without showing any symptoms of pharyngitis which further underlines that the likelihood to develop invasive disease among individuals colonized with *S. pyogenes* is very low. This and the high mortality rate of invasive diseases indicate that the adaptation of *S. pyogenes* to its human host (*S. pyogenes* is a strict human pathogen) has evolved on mucosal surfaces and that the very rare invasive state is accidental with weak or no evolutionary impact.

Starting in the 1980s, a marked increase of invasive *S. pyogenes* infections was recorded in Europe and the USA (Kaplan, 1991), and since then M1 strains have been the most frequently serotype recovered from patients with invasive disease in this part of the world and in Australia (O’Grady et al., 2007; Nelson et al., 2016; Gherardi et al., 2018). However, it is important to stress that in these countries M1 is the most common serotype also in superficial pharyngitis, indicating that M1 bacteria have developed selection advantage in the ecological niche most important for *S. pyogenes*. SIC was discovered in the M1 strain (AP1) used in this study, and during the 25 years we have used AP1 bacteria and the SIC protein in different experiments *in vitro, ex vivo* and *in vivo*, we have not observed any variations in size or sequence of the SIC protein or its gene (*sic*). This is not surprising since our experiments have not put a selective pressure on *sic*. However, analysis of strains from two consecutive M1 epidemics revealed that each epidemic wave was composed of several new SIC variants and that natural selection had contributed to the variation (Stockbauer et al., 1998). In a follow-up study, the same group analyzed 240 random M1 isolates from patients with pharyngitis and found 58 SIC variants. This ratio of variants was almost identical to what was found for invasive M1 isolates, showing that penetration into sterile sites does not drive selection (Hoe et al., 1999). Together with the observation that repeated intraperitoneal passage in mice did not generate new SIC variants (Stockbauer et al., 1998), and the low number of invasive compared to non-invasive infections discussed above, this demonstrates that *sic* selection takes place in the pharynx mucosa.

It is not clear what evolutionary pressure drives the rapid and extensive selection of new SIC variants, but a possible explanation could be found in a unique property of SIC; its capability to inhibit a large number of antimicrobial proteins and peptides which are important components of innate immunity and with bactericidal activity against *S. pyogenes* (Fernie-King et al., 2002; Frick et al., 2003; Egesten et al., 2007). The complement system, and the contact system (Frick et al., 2006), are two additional branches of innate immunity which when activated also generate antibacterial peptides (C3a of complement and fragments of H-kininogen of the contact system) that kill *S. pyogenes* but are inhibited by SIC (Frick et al., 2011). C3a is a pro-inflammatory peptide and activation of the contact system results in the release of bradykinin, which is also a potent pro-inflammatory peptide. SIC reduces contact activation and thereby bradykinin release (Åkesson et al., 2010). This means that SIC will protect the bacteria against various antibacterial proteins and peptides, and because of the interference with the complement and contact systems, the protein will simultaneously have an anti-inflammatory effect. However, in pharyngitis streptococcal colonization has induced an inflammatory host response causing vascular leakage and the recruitment of leukocytes, including monocytes, antibacterial proteins, and peptides, and complement and contact proteins, creating an environment with considerable evolutionary pressure and a hotbed for new SIC variants. In addition, several studies have found that spontaneous mutations in genes encoding the CovR/S two-component global regulatory system may transform M1 bacteria into a more invasive genotype [for more references see (Cole et al., 2011)]. For the present investigation it is striking that among the genes upregulated by CovR/S mutations are the genes encoding SIC and M1 protein. Significant amounts of SIC (Åkesson et al., 1996) and M1 (Åkesson et al., 1994) are produced and released by M1 bacteria into the growth medium, suggesting that the invasive genotype could produce even higher amounts. It is therefore noteworthy that released M1 protein form complexes with fibrinogen in blood and plasma which activate neutrophils to release heparin-binding protein (Herwald et al., 2004), a potent inducer of vascular leakage (Gautam et al., 2001).

Massive inflammation and vascular leakage are characteristic symptoms in severe invasive *S. pyogenes* disease. In these patients isolates of the M1 serotype are the most frequent, and they release M1 protein and SIC. The demonstration here that SIC, like M1, has powerful pro-inflammatory properties, indicate that both proteins contribute to the particular virulence of the M1 serotype and the pathophysiology of a very serious clinical condition. The results also identify SIC and its interactions with monocytes as new potential therapeutic targets.

### Limitations of the study

As a potential caveat of our work presented here, one could consider the used concentration of SIC. Since we found 5 μg/mL in THY growth medium, all our experiments were performed with this concentration. We added additional information about lower concentrations (Figure 3I), decreasing the protein concentration down to 1.25 μg/mL. This still resulted in a significant reponse in the monocytes. However, future experiments are needed to determine the SIC concentration released by *S. pyogenes* during ongoing infections.

The use of CD14 + magnetic beads for the isolation of a specific monocyte subpopulation from human blood could possibly affect the CD14 + receptor. Thus, a potential false lower response in primary monocytes cannot be excluded.

### Resource availability

#### Lead contact

Further information and requests for resources and reagents should be directed to and will be fulfilled by the lead contact, Ariane Neumann (ariane.neumann@med.lu.se.)

#### Materials availability

No unique reagents have been generated and there are no restrictions on availability.

#### Data and code availability

No custom code, software, or algorithm was used here.

## Methods

All methods can be found in the accompanying Transparent methods supplemental file.

## Supplemental information

Document S1. Transparent methods and Figure S1
