# Supplementary material for: Streptococcal protein SIC activates monocytes and induces inflammation
Source: iScience. 2021 Mar 20;24(4):102339. doi: 10.1016/j.isci.2021.102339 (PMC8027542; doi:10.1016/j.isci.2021.102339)
Supplement: Document S1. Transparent methods and Figure S1 [file mmc1.pdf]

iScience, Volume *24*

Supplemental information

Streptococcal protein SIC activates monocytes and induces inflammation

Ariane Neumann, Lotta Happonen, Christofer Karlsson, Wael Bahnan, Inga-Maria Frick, and Lars Björck

Supplemental items

Supplementary figure + legend

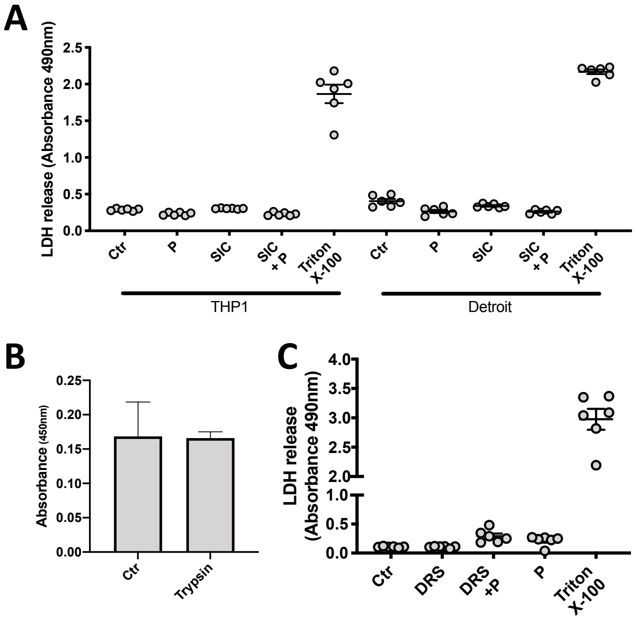

Supplementary figure 1. M1-SIC and M55-SIC display no cytotoxic effect on host cells. A) related to Fig. 3, B) related to Fig. 7, C) related to Fig. 8

Supplementary figure 1. M1-SIC and M55-SIC display no cytotoxic effect on host cells.

A: THP1 and Detroit 562 cells were incubated with 5 µg/ml SIC +/- 2.5% plasma (P) for 18 h and LDH release was detected by reading absorbance at 490 nm. Triton X-100 served as lysis control.

B:CD14$^+$ cells were incubated with 100 µg/ml Trypsin for 30 min. LDH release was measured at 450 nm. No increase of LDH release was detected after Trypsin treatment.

C: THP1 cells were incubated with 5 µg/ml of distantly related SIC (DRS) +/- 2.5% plasma (P) and release of LDH was measured. Triton X-100 served as lysis control.

Transparent Methods

Bacterial strains, purification of proteins and antibodies

S. pyogenes strain AP1 (40/58) serotype M1 was from WHO Collaborating Centre for references and research on Streptococci, Institute of Hygiene and Epidemiology, Prague, Czech Republic. The ΔSIC strain was generated from AP1 as described in (Frick *et al.*, 2003) by

allelic replacement mutagenesis strategy. *S. pyogenes* strain W38 (GT 71-154) serotype M55 was a kind gift of the late Dr. Wannamaker. Bacteria were grown in TH medium + 2% yeast at 37 °C overnight and 150 µg/ml Kanamycin was added for the ΔSIC strain. Supernatants from the cultures were collected at an OD_{620nm} of 0.5. M1-SIC and M55-SIC from *S. pyogenes* strains AP1 and W38, respectively, were purified by precipitation of the culture medium with 30% ammonium sulphate, followed by an ion-exchange chromatography on a MonoQ column (Amersham). Purity of M1-SIC was analysed via quantitative MS analysis. For infection experiments, overnight cultures of the bacteria were spun down and resuspended in RPMI medium (Gibco) + 10 % FCS (Thermo). Bacteria were grown until OD_{620nm} of 0.5, the supernatant was collected, sterile filtered, concentrated and stored at -20 °C until further usage. For analysis of SIC expression in the growth medium, proteins were precipitated with TCA and the protein concentration was analysed by direct ELISA.

Mouse monoclonal IgG antibodies against p38 MAP kinase and phosphorylated p38 MAP kinase were from Cell signalling, and polyclonal rabbit IgG antibodies against TLR2 and CD14 were from Invivogen.

Cell culture

THP1 cells (ATCC® TIB-202™) were cultivated in RPMI + 10% FBS and 1% Anti/Anti (all from Thermo). THP1 XBlue $CD14^+$ cells (Invivogen) were cultivated in RPMI-FBS-Anti/Anti with 250 µg/ml G418 (Invivogen) and 200 µg/ml Zeocin (Invivogen). Detroit 562 cells (ATCC® CCL-138™) were grown in DMEM medium (Thermo) with 10% FBS and 1% Anti/Anti. All cell lines were used in low passage numbers. Primary $CD14^+$ monocytes (ethical permit 2020:24, Lund University) were isolated by density gradient centrifugation and magnetic bead separation. Briefly, 10 ml of leukocyte concentrate were diluted 1:1 with 0.9% NaCl. 20 ml of that mixture was layered onto 20 ml of Lymphoprep™(AxisShield) and centrifuged for 20 min at 700 x g without brake. Erythrocytes were lysed with H_2O for 15 s. Purified PBMCs were resuspended in MACS buffer and CD14 microbeads (both Miltenyi Biotec) were added. Cells were then sorted with a LS column using a MACS separator (both Miltenyi Biotec). After the separation, cells were resuspended in RPMI without supplements (Thermo) and cell numbers were adjusted to respective experiments. For all experiments, RPMI supplemented only with 10% FBS was used. Pre-incubation with 100 µg/ml trypsin was performed in un-supplemented RMPI, cells were subsequently spun and resuspended in RMPI + 10% FBS. For inhibition studies, cells were pre-incubated with blocking IgG antibodies or IgGFc fragments: human IgG1 and Fc fragments (in house; 10 µg/ml), Pab-hTLR2 (20 µg/ml)

and anti-CD14 (10 µg/ml) both from Invivogen. Pre-incubation was performed for 15-60 min at 37 °C 5% CO_2, prior to stimulation with purified SIC.

Detection of inflammatory cytokines and chemokines

Cells were seeded in 96-well plates with $5*10^5$ cells/ml and incubated with 5 µg/ml purified SIC, 2.5% plasma or both for 2 h at 37 °C and 5% CO2. After incubation, the supernatant was collected and stored at -80 °C until further analysis. For an overall cytokine profiling, samples were analysed using the Bio-Plex Pro™ Human Cytokine 27-plex Assay (Biorad). Additionally, the secretion of TNFα, was analysed according to the manufacturer´s recommendation with a human TNFα uncoated ELISA (Invitrogen).

Western blot analysis

For detection of p38 MAP kinase phosphorylation, $2*10^6$/ml cells were seeded in 24-well plates with 500 µl per well and stimulated with SIC (5 µg/ml), SIC fragments (I,II, III; 5 µg/ml), DRS (5 µg/ml) and/or plasma (2.5%) or bacterial supernatant for 18 h at 37 °C and 5% CO_2. RPMI medium was used as a negative control. Cells were washed with 1 X PBS and lysed using RIPA buffer (Thermo) with a protease inhibitor mix (Thermo) for 5 min on ice. Cell lysates were boiled for 5 min at 95 °C with 4X Lämmli buffer and 10 µl samples were loaded on 4-20% TGX gel (Biorad) and run for 50 min at 150 V. Samples were transferred onto Trans-Blot® Turbo™ Mini PVDF membranes (Biorad) for 5 min at 25 V and 2.5 mA. Membranes were incubated overnight with primary antibodies (diluted 1:1000 in PBS-Tween with 5% BSA) against phosphorylated p38 MAP kinase (Phospho-p38 MAPK Thr180/Tyr182) or p38 MAP kinase (both from Cell signalling) at 4 °C, antibodies were diluted 1:1000 in PBS-Tween with 5% BSA. Secondary antibodies (goat anti-rabbit HRP-conjugated, Biorad) were diluted 1:5000 in PBS-Tween and membranes were incubated for 1-2 h at room temperature (RT). Membranes were visualised using Clarity Max™ Western ECL Blotting Substrate (Biorad). Band intensities were analysed using ImageJ software, normalising the phospho-p38 band to the according p38 band.

NF-κB activation in THP1 XBlue $CD14^+$ cells

THP1 XBlue $CD14^+$ cells were resuspended in RPMI + 10% FBS and seeded with $5*10^6$/ml cell in 96-well plates with 100 µl cells per well. Cells were stimulated according to manufacturer´s recommendation with 10 µl stimuli (5 µg/ml purified SIC or DRS, 2.5 % plasma, 5 µg/ml M1- SIC fragments I, II and III, 10 ng/ml LPS, 10 µl bacterial growth medium)

for 18 h at 37 °C and 5% CO_2. For removal of surface proteins, cells were pre-incubated for 30 min with 100 µg/ml trypsin in un-supplemented RPMI, spun down and resuspended in RPMI + 10% FBS to continue with respective stimuli. To analyse possible binding of SIC to monocyte surface proteins THP1 cells were pre-incubated with IgG_1, F_c fragment or antibodies against TLR2 or CD14 for 20 min, followed by the addition of the various stimuli. 20-50 µl cell supernatant was transferred into a new 96-well plate containing 150-180 µl QuantiBlue solution (Invivogen) per well and incubated for 1-4 h at 37 °C. Changes in colour were measured at 655 nm.

Interaction studies with fluorescently labelled protein SIC

Purified protein SIC was labelled with an Alexa Fluor® succinimidyl ester 633 overnight. Excessive dye was removed, and the protein concentration after labelling was determined. THP1 cells were seeded in a µ-slide (ibidi) at a concentration of $2*10^6$/ml. Labelled SIC (5 µg/ml) was added and samples were incubated for 1 h at 37 °C. Live imaging was performed using a Nikon Eclipse TE300 fluorescence microscope with a PlanFluor 40X/0.60 NA objective (Bergman Labora, Lyckeby, Sweden). For quantification, samples were analysed using a BD Accuri™ C6 flow cytometer. Pre-incubation of cells with 20 µM Cytochalasin D for 30 min was used to block possible uptake/binding of the protein. Additionally, uptake/binding was blocked by incubation of the cells and labelled SIC for 1 h on ice. Cells were washed with 1 X PBS to remove excessive and unbound protein and dye before imaging and FACS analysis.

Surface plasmon resonance binding studies

SPR experiments were performed using a Biacore T100 (GE Healthcare) at 25 °C. TLR2 or CD14 were immobilised on a CM5 chip; as running buffer 100 mM HEPES, 150 mM NaCl, 0.05% Surfactant P20, pH 7.4 (GE Healthcare) was used at 10 µl/min. Immobilisation of TLR2 resulted in 250.1RU and CD14 in 237.7 RU. SIC concentrations prepared in running buffer were the following: 2.5 µg/ml, 5 µg/ml, 10 µg/ml, 20 µg/ml, 40 µg/ml, 80 µg/ml. Samples were run in flow cell 1,2 with one regeneration. 10 mM Glycine HCl, pH 2.5 was used for the regeneration with 30 sec contact time and 0 sec stabilisation period. Contact time with the analyte for 120 sec, dissociation time 180 sec. Binding affinities of SIC to either TLR2 or CD14 were calculated from rate constants obtained from fitting the data to a 1:1 Langmuir binding model. Average K_D values were displayed in the graphs.

Sample preparation for mass spectrometry

THP1 were resuspended in RPMI medium without FBS and adjusted to $5*10^6$/ml. Cells were then incubated with 100 µg/ml trypsin for 30 min, spun for 5 min and resuspended with RPMI + 10% FBS. Cells were then lysed with 300 µl RIPA buffer (Thermo) with a protease inhibitor mix (Thermo) for 5 min on ice. For analysis, 100 µg of cell lysates in triplicates were precipitated with acetone followed by an ethanol wash. The precipitated proteins were mixed with 8 M urea and 100 mM ammonium bicarbonate, and the cysteine bonds were reduced with 5 mM TCEP (37 °C for 90 min) and alkylated with 10 mM iodoacetamide (22 °C for 30 min). The samples were diluted with 100 mM ammonium bicarbonate to a final urea concentration of below 1.5 M, and sequencing grade trypsin (Promega) was added for protein digestion (37 °C for 16 h). The samples were acidified (to a final pH 3.0) with 10% formic acid, and the peptides purified with Solaµ HRP columns according to the manufacturer's instructions (Thermo). Peptides were dried in a speedvac and reconstituted in 2% acetonitrile, 0.2% formic acid prior to mass spectrometric analyses.

Liquid chromatography tandem mass spectrometry (LC-MS/MS)

All peptide analyses were performed on Q Exactive HF-X mass spectrometer (Thermo Scientific) connected to an EASY-nLC 1200 ultra-high-performance liquid chromatography system (Thermo Scientific). Peptides were loaded onto an Acclaim PepMap 100 C18, 3 µm, 100Å pre-column (ID 75µm x 2 cm) and separated on an EASY-Spray column (Thermo Scientific; ID 75µm x 50 cm, column temperature 45 °C) operated at a constant pressure of 800 bar. A linear gradient from 4 to 45% of 80% acetonitrile in aqueous 0.1% formic acid was run for 125 min at a flow rate of 350 nl min-1.

For DDA MS, one full MS scan (resolution 60000 @ 200 m/z; mass range 390–1 210m/z) was followed by MS/MS scans (resolution 15000 @ 200 m/z) of the 15 most abundant ion signals. The precursor ions were isolated with 2 m/z isolation width and fragmented using HCD at a normalized collision energy of 30. Charge state screening was enabled, and precursors with an unknown charge state and a charge state of 1 and above 6 were rejected. The dynamic exclusion window was set to 10 s. The automatic gain control was set to 3e6 and 1e5 for MS and MS/MS with ion accumulation times of 110 ms and 60 ms, respectively. The intensity threshold for precursor ion selection was set to 1.7e4.

For data independent acquisition (DIA) MS, one full MS scan (resolution 60000 @ 200 m/z; mass range from 390 to 1210m/z) was followed by 32 MS/MS full fragmentation scans (resolution 30000 @v200 m/z) using an isolation window of 26 m/z (including 0.5 m/z overlap

between the previous and next window). The precursor ions within each isolation window were fragmented using HCD at a normalized collision energy of 30. The automatic gain control was set to 3e6 and 1e6 for MS and MS/MS with ion accumulation times of 100 ms and 120 ms, respectively.

Mass spectrometry data analysis

All raw data were converted to gzipped and NumPressed (Teleman *et al.*, 2014) mzML using the tool MSconvert from the ProteoWizard, v3.0.5930 suite (Chambers *et al.*, 2012). All data analyses were stored and managed using openBIS (Barillari *et al.*, 2016). DDA acquired spectra were analysed using the search engine X! Tandem (2013.06.15.1-LabKey, Insilicos, ISB) (Craig and Beavis, 2003) and OMSSA (Geer *et al.*, 2004) against an in-house compiled database (Quandt *et al.*, 2014) containing the *Homo sapiens* Uniprot reference proteome (ID UP000005640) with 74823 entries and an equal number of reverse decoys, follow by a Trans-Proteomic Pipeline (TPP v4.7 POLAR VORTEX rev 0, Build 201403121010) using PeptideProphet (Keller *et al.*, 2002) and false discovery rate (FDR) estimation with Mayu (v1.7) (Reiter *et al.*, 2009). The spectral library was built with TPP Spectrast using the filtered peptide spectrum matches (PSMs) with all retention times converted to iRTs (Lam *et al.*, 2008). All DIA MS runs were aligned using TRIC (Röst *et al.*, 2016) to produce a final data matrix with 1% FDR at peptide level and 10% FDR at protein level.

Supplemental References

Barillari, C. *et al.* (2016) 'OpenBIS ELN-LIMS: An open-source database for academic laboratories', *Bioinformatics*. doi: 10.1093/bioinformatics/btv606.

Chambers, M. C. *et al.* (2012) 'A cross-platform toolkit for mass spectrometry and proteomics', *Nature Biotechnology*. doi: 10.1038/nbt.2377.

Craig, R. and Beavis, R. C. (2003) 'A method for reducing the time required to match protein sequences with tandem mass spectra', *Rapid Communications in Mass Spectrometry*. doi: 10.1002/rcm.1198.

Frick, I. M. *et al.* (2003) 'SIC, a secreted protein of Streptococcus pyogenes that inactivates antibacterial peptides', *Journal of Biological Chemistry*. doi: 10.1074/jbc.M301995200.

Geer, L. Y. *et al.* (2004) 'Open mass spectrometry search algorithm', *Journal of Proteome Research*. doi: 10.1021/pr0499491.

Keller, A. *et al.* (2002) 'Empirical statistical model to estimate the accuracy of peptide identifications made by MS/MS and database search', *Analytical Chemistry*, 74(20), pp. 5383–

5392. doi: 10.1021/ac025747h.

Lam, H. *et al.* (2008) 'Building consensus spectral libraries for peptide identification in proteomics', *Nature Methods*. doi: 10.1038/nmeth.1254.

Quandt, A. *et al.* (2014) 'Using synthetic peptides to benchmark peptide identification software and search parameters for MS/MS data analysis', *EuPA Open Proteomics*. European Proteomics Association (EuPA), 5, pp. 21–31. doi: 10.1016/j.euprot.2014.10.001.

Reiter, L. *et al.* (2009) 'Protein identification false discovery rates for very large proteomics data sets generated by tandem mass spectrometry', *Molecular and Cellular Proteomics*. doi: 10.1074/mcp.M900317-MCP200.

Röst, H. L. *et al.* (2016) 'TRIC: An automated alignment strategy for reproducible protein quantification in targeted proteomics', *Nature Methods*. doi: 10.1038/nmeth.3954.

Teleman, J. *et al.* (2014) 'Numerical compression schemes for proteomics mass spectrometry data', *Molecular and Cellular Proteomics*. doi: 10.1074/mcp.O114.037879.
